# Controllable Preparation and Optimisation of Bi_4_O_5_Br_2_ for Photocatalytic Reduction of CO_2_ to CO

**DOI:** 10.3390/ma18235442

**Published:** 2025-12-02

**Authors:** Xiaolong Cai, Baiquan Jing, Rong Li, Yongbo Ma, Baowei Cao, Yunhua Xu

**Affiliations:** 1 College of New Energy, Yulin University, Yulin 719000, China; 62534abc@163.com; 2 College of Chemistry and Chemical Engineering, Yulin University, Yulin 719000, China; jbq011016@163.com (B.J.); lirong_w0313@163.com (R.L.); 13891229998@163.com (B.C.); xuyunhua0726@163.com (Y.X.); 3 School of Material Science and Engineering, Xi’an University of Technology, Xi’an 710048, China

**Keywords:** photocatalysis, Bi_5_O_4_Br_2_, various morphologies, CO_2_ reduction

## Abstract

The use of photocatalytic CO_2_ reduction as a green technology has attracted the attention of scholars. Nevertheless, the lower visible-light utilisation and photocatalytic efficiency of catalysts remain a challenge. In this work, Bi_x_O_y_Br_z_ photocatalysts were synthesised using a hydrothermal method by adjusting the molar ratio of Bi(NO_3_)_3_·5H_2_O and C_19_H_42_BrN (Bi:Br ratio) and the pH value of the precursor solution. The obtained samples were characterised, and the CO_2_ reduction performance was tested. The results showed that the phase composition for most of the samples was Bi_4_O_5_Br_2_, and BiOBr or Bi_5_O_7_Br was also confirmed in a small number of samples. Owing to the effects of pH and the Bi:Br ratio on the reaction process, BiOBr→Bi_4_O_5_Br_2_→Bi_5_O_7_Br transformation occurred. Acidic conditions are conducive to the formation of BiOBr. In alkaline environments, bismuth-rich Bi_4_O_5_Br_2_ or even Bi_5_O_7_Br easily forms. Bi_4_O_5_Br_2_ has self-assembled microsphere and irregular polyhedron morphologies. The polyhedron Bi_4_O_5_Br_2_ results in CO and CH_4_ yields of 10.34 μmol·g^−1^·h^−1^ and 1.86 μmol·g^−1^·h^−1^ in CO_2_ reduction, respectively. Although the microsphere Bi_4_O_5_Br_2_ has a maximum light absorption wavelength of 438 nm, the polyhedron Bi_4_O_5_Br_2_ has the best photocatalytic CO_2_ reduction performance and CO selectivity. This work describes the controllable preparation of Bi_4_O_5_Br_2_ at various pH values and Bi:Br ratios and the optimisation of its photocatalytic performance.

## 1. Introduction

The excessive emission of carbon dioxide (CO_2_) has caused the greenhouse effect, which has led to global climatic warming [1,2,3]. In this case, CO_2_ reduction is urgent. CO_2_ reduction into value-added chemicals is considered as one of the most effective pathways for attaining carbon neutrality [2]. Carbon monoxide (CO) is an important raw material in Fischer–Tropsch synthesis [4]. Therefore, reducing CO_2_ to CO is important for alleviating the greenhouse effect and energy crisis. Because CO_2_ molecules are extremely stable, their reduction process needs to overcome a large energy barrier [1,5]. Compared with thermocatalysis and electrocatalysis, photocatalysis has the advantages of lower energy consumption and lower cost [6]. Solar-driven photocatalytic CO_2_ reduction to CO is proposed as a promising, green method [7,8]. However, the lower visible-light utilisation and photocatalytic efficiency of catalysts limit the wider application of this method. Hence, the development of efficient photocatalyst materials remains a challenge for CO_2_ reduction.

Bismuth oxybromide (Bi_x_O_y_Br_z_) semiconductor photocatalysts, such as BiOBr, Bi_4_O_5_Br_2_ and Bi_5_O_7_Br, have attracted the attention of many researchers due to their excellent photocatalytic performance [5,9,10]. Among them, Bi_4_O_5_Br_2_ has a band gap that is favourable for the formation of photogenic charge carriers [11]. Additionally, Bi_4_O_5_Br_2_, as a bismuth-rich oxybromide, possesses a unique layered crystal structure, where the charge density around the [Bi-O] layer is greater than that around the [Br-Br] layer [9,12]. This difference in the electron density distributions between the [Bi-O] and [Br-Br] layers generates an increase in the internal electric field intensity, which can improve the charge separation efficiency. H. Cai et al. reported that oxygen vacancy-mediated ultrathin Bi_4_O_5_Br_2_ nanosheets promoted the separation and transfer of piezoinduced charges in hydrogen peroxide production [13]. Y. Bai et al. synthesised two-dimensional Bi_4_O_5_Br_2_ nanosheets for the conversion of photocatalytic CO_2_ into CO, and their CO selectivity was greater than 99.5% [14]. Nevertheless, the utilisation of visible light and charge separation efficiency of Bi_4_O_5_Br_2_ are still lower.

To address the above problems, several modification strategies for catalysts have been proposed. Although many studies have focused on atom doping, heterostructure building, metal–organic frameworks (MOFs) and covalent organic frameworks (COFs) [3,5,8,15,16], the photocatalytic performance can also be optimised by adjusting and controlling the intrinsic structure of catalysts, such as their composition, morphology and size [1,14,17]. Generally, the presence of various morphologies, including one-dimensional nanotubes, two-dimensional nanosheets and three-dimensional self-assembling microspheres, affects the absorption, reflection and refraction of light. D. Mao et al. fabricated ultrathin Bi_4_O_5_Br_2_ nanotubes with abundant oxygen vacancies that extended the photo-response region and increased charge separation [2]. The band structure can be improved by means of regulating the ratio of Bi, O and Br, influencing the optoelectronic efficiency. Compared with BiOBr (2.68 eV), Bi-rich Bi_4_O_5_Br_2_ has a smaller band gap energy (2.25 eV) [9]. Furthermore, high surface-to-volume ratios, such as hollow and porous structures as well as nano size, are beneficial in increasing the number of active sites and also in improving CO_2_ absorption [18]. One study reported that nanosheets, layered microspheres and hollow spheres of Bi_4_O_5_Br_2_ were synthesised by a microemulsion method, where the concentration of the surfactant influenced the morphology and size of the catalyst [17]. In several studies [11,15,17,19,20], the effect of Bi_4_O_5_Br_2_ morphology on photocatalytic performance during the degradation of pollutants in wastewater has been revealed. Nevertheless, the effects of pH and the Bi:Br ratio of the precursor solution on the phase structure and morphology of Bi_x_O_y_Br_z_ are limited during CO_2_ reduction to CO in the literature.

The objective of this study was to explore the relationship between the structure and performance of photocatalysts. In this study, Bi_4_O_5_Br_2_ catalysts with various morphologies were prepared using a hydrothermal method by adjusting the pH and Bi:Br ratio of the precursor solution. The phase composition, structure and morphology of the acquired catalysts were characterised. The effects of pH and the Bi:Br ratio on the phase type and morphology of the catalysts were discussed, and the chemical composition and photoelectric, electrochemical and CO_2_ adsorptive properties of Bi_4_O_5_Br_2_ were evaluated. Furthermore, the yield and selectivity of photocatalysts for CO_2_ reduction into CO were investigated via gas chromatography.

## 2. Experimental Procedure

### 2.1. Synthesis of Bi_4_O_5_Br_2_

Bi_4_O_5_Br_2_ photocatalysts were synthesised using a hydrothermal method. First, Bi(NO_3_)_3_·5H_2_O (0.006, 0.012, 0.015, 0.018, 0.024 and 0.048 mol) was dissolved in 30 mL of glycerinum to obtain A solutions of varying concentrations. Further, C_19_H_42_BrN (0.006 mol) was added to the A solutions and stirred magnetically for 30 min until it uniformly mixed to form suspension solution B. To adjust the pH of the reaction mixture, NH_3_·H_2_O or hydrochloric acid was added to suspension solution B and subsequently stirred for 2 h to obtain C solution. The pH value of the solution was controlled at 5, 7, 9 and 11 by test paper. Furthermore, the C solution was transferred into a 100 mL reaction vessel, which was heated to 160 °C, maintained for 16 h, and then naturally cooled to room temperature. After the reaction, the precursors were precipitated via a centrifugal treatment. The acquired precursors were washed three times with deionised water or anhydrous ethanol. Finally, the washed precursors were dried at 60 °C for 8 h in a vacuum oven to obtain various samples. The molar ratios of Bi to Br were 1:1, 2:1, 2.55:1, 3:1, 4:1 and 8:1 at different pH values. Considering these reaction conditions, the resulting samples were designated as shown in Table 1.

### 2.2. Sample Characterisation

The phase composition of the samples was determined via X-ray diffraction (XRD, Bruker, Karlsruhe, Germany) with Cu Kα radiation, a step size of 0.02 and a 2θ of 0–80°. The microstructures and energy spectra of the samples were characterised via field emission scanning electron microscopy (SEM, σ-300, Carl Zeiss AG, Jena, Germany) at voltages of 10 kV and 20 kV. The light absorption performance of the prepared catalysts was determined via UV–visible diffuse reflectance spectroscopy (UV–visDRS) (Shimadzu, UV-2600, Kyoto, Japan). The chemical state of the catalyst surfaces was analysed through X-ray photoelectron spectroscopy (XPS, Nexsa G2, Thermo Fisher Scientific, Madison, WI, USA). Mott–Schottky curves of the synthesised samples were obtained on an electrochemical workstation (Shanghai Chenhua Instrument Co., Ltd., CHI760E, Shanghai, China). Further, the tests were performed with a standard three-electrode system with a working electrode, Ag/AgCl reference electrode and Pt counter electrode. The adsorption properties of the catalysts for N_2_ and CO_2_ were measured via a specific surface area analyser (Quantachrome, AutoSorb-IQ, Anton Paar QuantaTec Inc., Boynton Beach, FL, USA).

### 2.3. Photocatalytic Reactions

The light source was simulated using a xenon lamp with a wattage of 300 W (Beijing Zhongjiao Jinyuan Technology Co., Ltd., CEL-HXF300-T3, Beijing, China) equipped with a continuous reactor (100 mL). Firstly, 10 mg of the prepared catalyst was put into the abovementioned photoreactor. Second, deionised water (1 mL) and moderate CO_2_ gas were introduced into the photoreactor. Furthermore, the photocatalytic reduction reactions of CO_2_ were carried out with a simulated sunlight time of 1 h, temperature of 60 °C, pressure of about 0.2 MPa and a non-flowing CO_2_. Upon completion of the reaction, the gaseous mixture was transferred by argon purge to a gas chromatograph (Fuli Analytical Instruments Co., Ltd., GC9790II, Taizhou, China) for analysis. Separation was performed on an RB-WAX chromatographic column (30 m × 0.32 mm × 0.5 μm) held at 120 °C, with argon as the carrier gas at a flow rate of 1.0 mL/min. The components were detected by a thermal conductivity detector (TCD) and quantified using the external standard method based on peak areas.

## 3. Results and Discussion

### 3.1. Influence of pH and the Bi/Br Ratio on the Phase Composition

**Figure 1:**
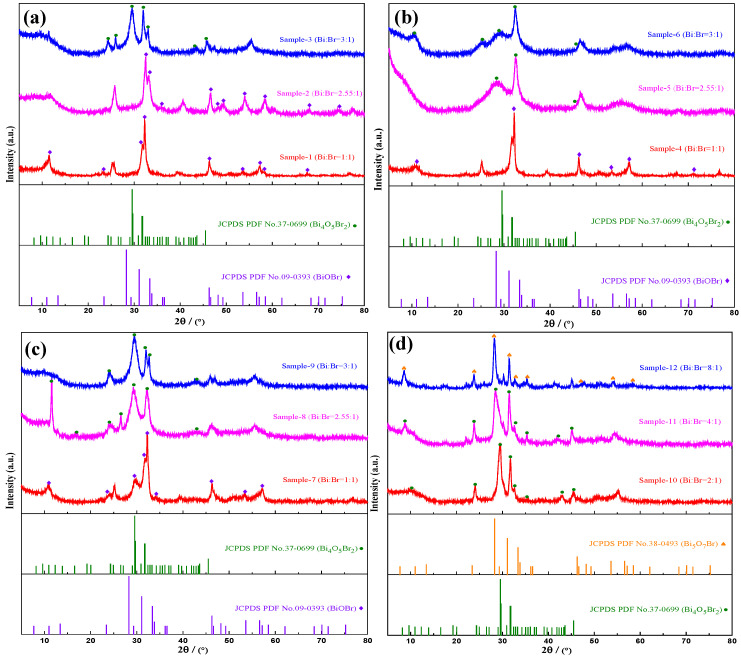
XRD spectra of the samples at different pH values and Bi/Br ratios: (**a**) pH = 5, (**b**) pH = 7, (**c**) pH = 9, (**d**) pH = 11.

Figure 1 shows the XRD patterns of all the samples at different pH values and Bi/Br ratios. The characteristic peaks observed in Figure 1a for sample-1 and sample-2 at 2θ values of 10.900°, 25.157°, 32.220°, 46.208° and 57.116° correspond to the (001), (101), (110), (200) and (212) crystal planes, respectively, compared with the standard PDF card (JCPDS 09-0393), which indicates that the phase type of both is BiOBr. The characteristic peaks of sample-3 at 2θ values of 10.948°, 24.946°, 29.548° and 31.671°, corresponding to the (101), (31-1), (11-3) and (402) crystal planes (JCPDS 37-0699), belong to the monoclinic crystal phase of Bi_4_O_5_Br_2_. This result indicates that Bi_4_O_5_Br_2_ crystals are obtained with Bi:Br = 3:1 at a solution pH of 5 and that phase transformation occurs from BiOBr to Bi_4_O_5_Br_2_ with increasing Bi/Br ratio. XRD patterns of the samples prepared under pH = 7 are presented in Figure 1b, where the phase structure of sample-4 is BiOBr, and both sample-5 and sample-6 have a phase composition of Bi_4_O_5_Br_2_. As the Bi/Br ratio increases, the intensity of the diffraction peak of Bi_4_O_5_Br_2_ gradually increases, and the peak shape becomes sharper, but there is no significant shift in the diffraction peak. The results suggest that the Bi_4_O_5_Br_2_ crystal is acquired at pH = 7 when the Bi:Br ratio reaches a value of 2.55:1. As described in Figure 1c, the phase composition of synthesised sample-7 is BiOBr with Bi/Br = 1:1, and those of prepared sample-8 and sample-9 are Bi_4_O_5_Br_2_ at pH = 9 and Bi/Br = 2.55:1 and Bi/Br = 3:1, respectively. This result is similar to that of the samples at pH = 7. The phase transformation of BiOBr to Bi_4_O_5_Br_2_ is also observed. Sample-10 and sample-11 at 2θ values of 28.292°, 31.066°, 46.275° and 53.617°, belonging to the (312), (004), (604) and (316) crystal planes, respectively, were determined to be the Bi_4_O_5_Br_2_ phase, and sample-12 had a phase composition of Bi_5_O_7_Br (Figure 1d). These results indicate that the Bi_4_O_5_Br_2_ crystals can be obtained at pH = 11 in solutions with Bi:Br ratios of 2:1 and 4:1. Additionally, as the Bi/Br ratio increases, Bi_5_O_7_Br crystals are found when the Bi:Br ratio reaches 8:1, which is accompanied by the phase transformation of B_4_O_5_Br_2_ to Bi_5_O_7_Br.

The XRD results of the four groups of samples indicate that the phase composition of most of the samples is Bi_4_O_5_Br_2,_ and a small portion of the samples consist of BiOBr or Bi_5_O_7_Br. Under the influence of pH and the ratio of Bi/Br, a phase transformation occurs from BiOBr→Bi_4_O_5_Br_2_→Bi_5_O_7_Br. When the solution is acidic, the BiOBr phase can be formed easily. When the solution is alkaline, the Bi_4_O_5_Br_2_ or even Bi_5_O_7_Br phase is more likely to be generated. This occurrs because the higher the pH value of the solution is, the more Br^−^ can be replaced by OH^−^ during the reaction, thereby generating bismuth-rich Bi_4_O_5_Br_2_ or even bismuth-rich and bromine-poor Bi_5_O_7_Br. When the pH is constant, as the ratio of Bi/Br in the reaction mixture increases, enriched Bi^3+^ can react with BiOBr to form Bi_4_O_5_Br_2_. Nevertheless, the excess Bi^3+^ reacts with Bi_4_O_5_Br_2_ to form Bi_5_O_7_Br. When the ratio of Bi/Br is constant, an increase in the pH of the solution will cause the phase composition of the samples to change from BiOBr→Bi_4_O_5_Br_2_→Bi_5_O_7_Br because more Br^−^ can be replaced by OH^−^ at higher pH values, resulting in phase transformation.

### 3.2. Influence of pH and the Bi/Br Ratio on the Morphology

**Figure 2:**
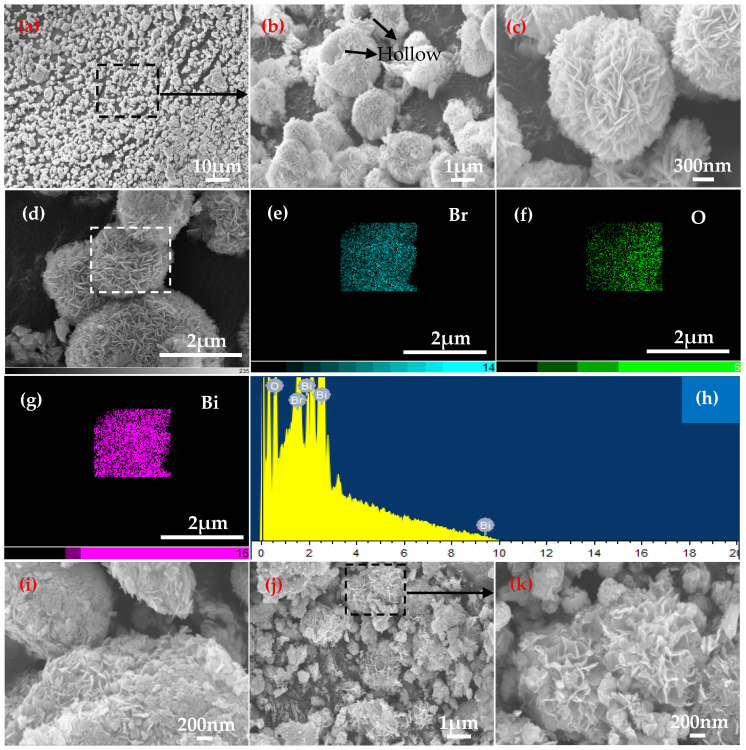
SEM images of the samples at pH = 5 for (**a**) Bi:Br = 1:1, (**b**) microstructure marked in (**a**), (**c**) enlarged morphology for (**b**), (**d**) SEM image, (**e**) Br, (**f**) O, (**g**) Bi, (**h**) EDS, (**i**) Bi:Br = 2.55:1, (**j**) Bi:Br = 3:1 and (**k**) enlarged detail marked in (**j**).

Figure 2 shows the SEM images and chemical compositions of samples prepared at pH = 5 and various Bi/Br ratios in the reaction solution. As shown in Figure 2a,b, the morphology of sample-1 is very regular and spherical, and several spheres present a hollow structure. Moreover, a magnified detail indicates that the regular sphere is formed by the self-assembly of many BiOBr nanosheets (Figure 2c). This hollow microsphere structure is conducive to increasing multiple reflections and scattering of light, thereby enhancing the light absorption capacity [18,21]. Figure 2d–g show the map scanning results for bismuthyl bromide on the sphere surface of sample-1. The distributions of Br, O and Bi are relatively uniform in bismuthyl bromide, where the diffraction peaks of the three elements are presented via EDS (Figure 2h). Table 2 provides the contents of the three elements by EDS point tests. The Bi/Br atomic ratio of 1.3:1 for sample-1 presents a 30% deviation from the experimental value of 1:1 (Table 1). When Bi:Br = 2.55:1, the phase composition of sample-2 remains BiOBr, but the morphology maintains tight and irregular microspheres (Figure 2i). Figure 2j shows SEM image of sample-3 prepared with Bi:Br = 3:1. The phase type changes from BiOBr to Bi_4_O_5_Br_2,_ and the morphology presents loose and irregular microspheres (Figure 2k). This morphology increases the pores and specific surface area, allowing more active surfaces and edges of Bi_4_O_5_Br_2_ to be exposed.

**Figure 3:**
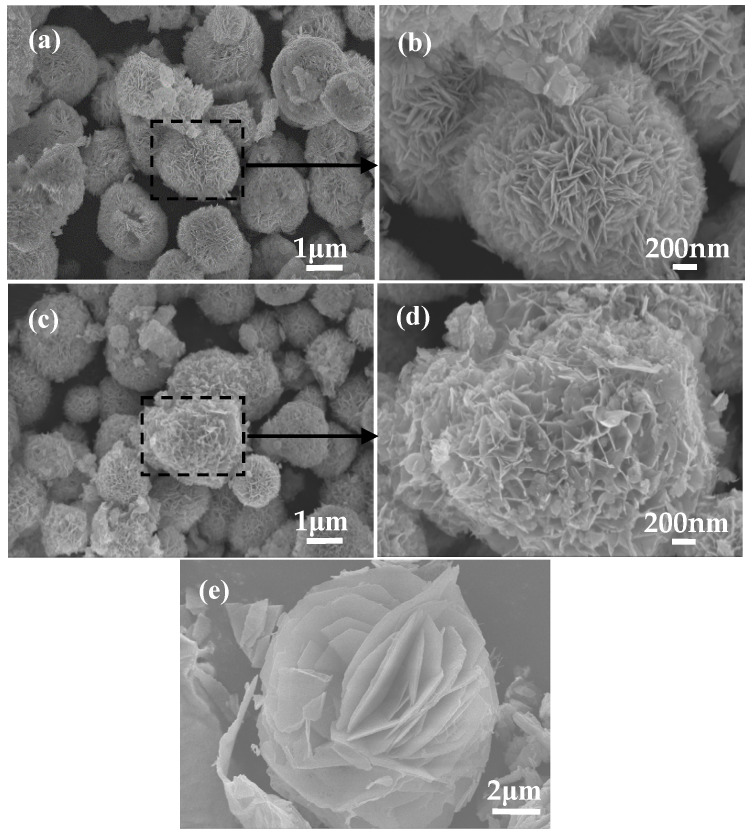
SEM images of the pH = 7 samples for (**a**) Bi:Br = 1:1, (**b**) magnified detail marked in (**a**), (**c**) Bi:Br = 2.55:1, (**d**) magnified detail marked in (**c**) and (**e**) Bi:Br = 3:1.

Figure 3 shows SEM images of samples synthesised at pH = 7 with various Bi/Br ratios in the reaction mixture. When the ratio of Bi:Br is 1:1, sample-4 possesses a morphology of self-assembled regular microspheres (Figure 3a), which are composed of many BiOBr nanosheets (Figure 3b). When the ratio of Bi:Br reached a value of 2.55:1, sample-5 presented loose and relatively regular microspheres composed of many thin Bi_4_O_5_Br_2_ nanosheets (Figure 3c). Compared with those in sample-4 and sample-5, the Bi_4_O_5_Br_2_ in sample-6 with microspheres are looser (Figure 3b,d,e). The reason for the formation of the spherical morphology is that the nucleation rate of Bi_4_O_5_Br_2_ can be accelerated with increasing concentration of bismuth nitrate in the solution [22].

**Figure 4:**
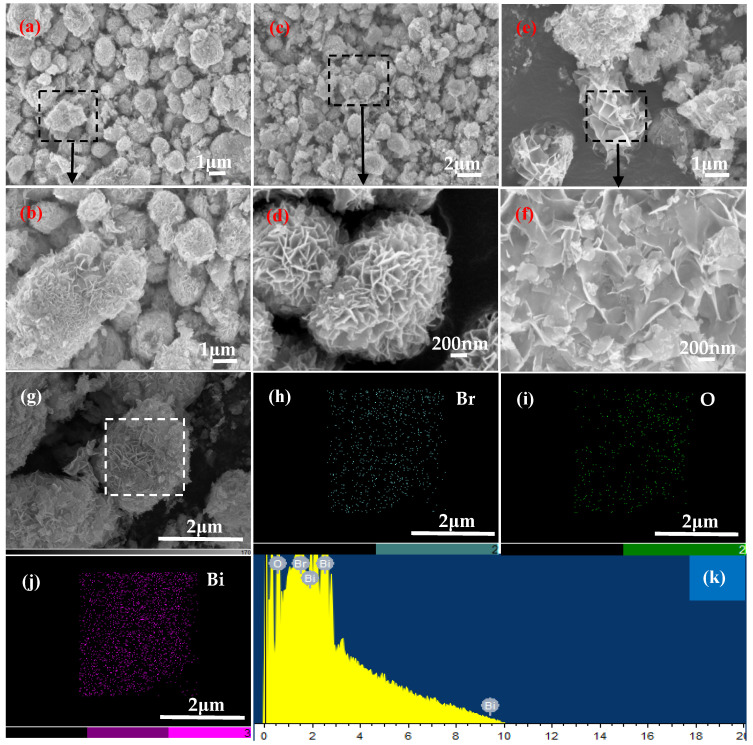
SEM images of the pH = 9 samples for (**a**) Bi:Br = 1:1, (**b**) enlarged detail tagged in (**a**), (**c**) Bi:Br = 2.55:1, (**d**) enlarged detail tagged in (**c**), (**e**) Bi:Br = 3:1, (**f**) magnified detail tagged in (**e**), (**g**) SEM image, (**h**) Br, (**i**) O, (**j**) Bi and (**k**) EDS.

SEM images of samples obtained at pH = 9 and various Bi/Br ratios are shown in Figure 4. Sample-7 presents irregular and loose microspheres and polyhedrons, which are formed by the self-assembly of many thin BiOBr nanosheets (Figure 4a,b). Sample-8 has relatively regular and loose microspheres self-assembled from Bi_4_O_5_Br_2_ nanosheets (Figure 4c,d). As shown in Figure 4e,f, most of the Bi_4_O_5_Br_2_ exhibited a very loose and irregular polyhedral morphology. Compared with the samples obtained at pH = 5 and pH = 7, Bi_4_O_5_Br_2_ is more likely to form loose and irregular polyhedrons in an alkaline environment (pH = 9). The polyhedral morphology of Bi_4_O_5_Br_2_ in sample-9 has larger pores than that of sample-8, leading to an increase in the number of active surfaces. Additionally, the map scanning result of sample-8 reveals that the distributions of Br, O and Bi are uniform in Bi_4_O_5_Br_2_ (Figure 4g–k).

**Figure 5:**
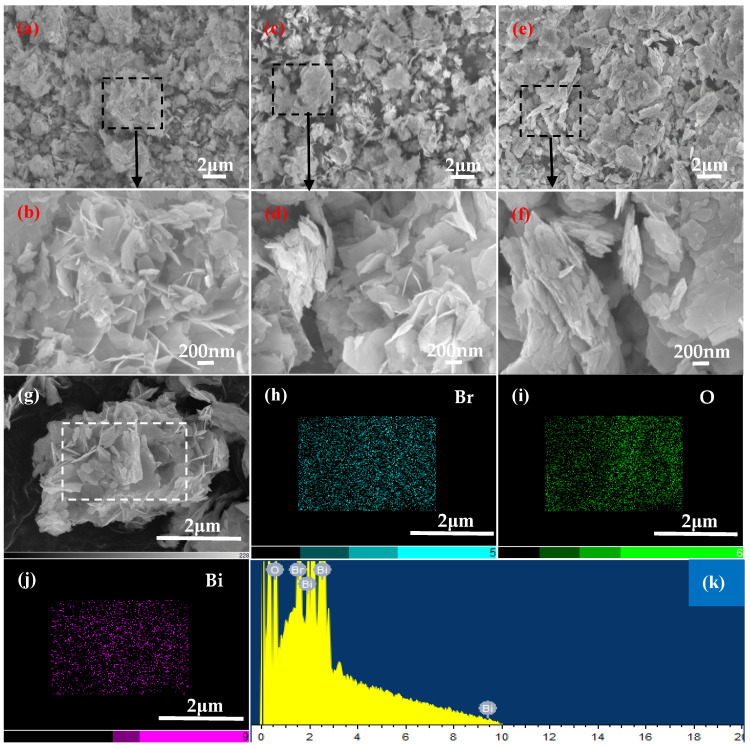
SEM images of the pH = 11 samples for (**a**) Bi:Br = 2:1, (**b**) enlarged detail tagged in (**a**), (**c**) Bi:Br = 4:1, (**d**) magnified detail tagged in (**c**), (**e**) Bi:Br = 8:1, (**f**) magnified detail tagged in (**e**), (**g**) SEM image, (**h**) Br, (**i**) O, (**j**) Bi and (**k**) EDS.

The SEM images of samples obtained at pH = 11 and various Bi/Br ratios are shown in Figure 5, where the phase composition of sample-10 and sample-11 is Bi_4_O_5_Br_2_ (Figure 1d), and the morphology of both reveal loose and irregular polyhedrons composed of numerous Bi_4_O_5_Br_2_ nanosheets (Figure 5a–d). Evidently, the distribution of Bi_4_O_5_Br_2_ nanosheets in sample-11 is looser than that in sample-10. Furthermore, the Bi/Br atomic ratio which is approximately 4.4:1 for sample-11 (Table 2), approaches the experimental value of 4:1 (Table 1). Nevertheless, sample-12, with a phase composition of Bi_5_O_7_Br, has a relatively dense lamellar morphology (Figure 5e,f). The map scanning result of sample-11 reveals that the distributions of Br, O and Bi are uniform in Bi_4_O_5_Br_2_ (Figure 5g–k). Furthermore, the contents of Bi, O and Br are 26.27 at.%, 70.35 at.% and 3.38 at.%, respectively. The Bi/Br atomic ratio has a value of approximately 7.8:1 (Table 2), which is lower than the experimental value of 8:1 (Table 1).

The phase composition and structure of the catalysts affect their photocatalytic performance. Therefore, it is necessary to regulate and optimise the phase composition and structure of catalysts. Although the microsphere, nanosheet and nanotube morphologies of BiOBr, Bi_4_O_5_Br_2_ and Bi_5_O_7_Br have been synthesised and reported in several studies [5,6,7,8,11,12,13,14,17,21,23], the regulation of phase composition and morphology of these catalysts remains a challenge. This work investigated the controllable preparation of BiOBr, Bi_4_O_5_Br_2_ and Bi_5_O_7_Br and the regulation of their morphology. Based on the above results, it can be deduced that the Bi/Br ratio affects the phase composition of the samples, whereas pH affects the morphology of the catalysts. Under acidic conditions, the relatively regular microsphere morphology is dominant (Figure 2). Under alkaline conditions, irregular polyhedron or even nanosheet shapes were obtained (Figure 4 and Figure 5).

### 3.3. Surface XPS Spectra

**Figure 6:**
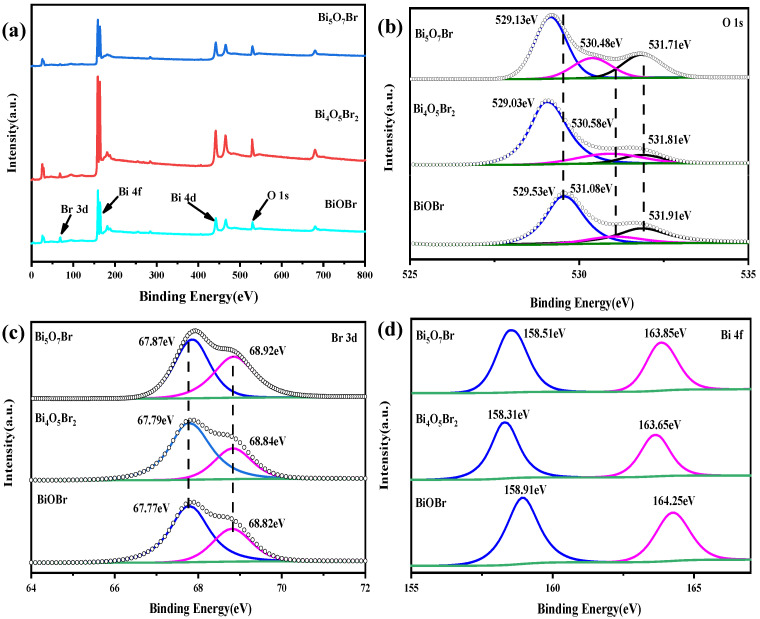
(**a**) XPS survey spectra and high-resolution spectra for (**b**) O 1s, (**c**) Br 3d and (**d**) Bi 4f of BiOBr, Bi_4_O_5_Br_2_ and Bi_5_O_7_Br.

To further evaluate the chemical state, the surface of samples was detected via XPS. The total XPS spectra of sample-2, sample-11 and sample-12 reveal that the prepared BiOBr, Bi_4_O_5_Br_2_ and Bi_5_O_7_Br are composed of Br, O and Bi, respectively (Figure 6a). Figure 6b–d show the high-resolution XPS spectra for the BiOBr, Bi_4_O_5_Br_2_ and Bi_5_O_7_Br. Furthermore, the O 1 s spectrum (Figure 6b) has three asymmetric peaks with band energies of 529.03 eV, 530.58 eV and 531.81 eV, corresponding to the lattice oxygen, oxygen vacancy and hydroxyl groups in Bi_4_O_5_Br_2_, respectively [2,7]. However, the two main peaks at 529.53 eV and 531.91 eV are obvious in BiOBr, but the oxygen vacancy signal is almost non-existent [5,9]. In Bi_5_O_7_Br, the band energies at 529.13 eV, 530.48 eV and 531.71 eV confirmed the presence of lattice oxygen, oxygen vacancy and hydroxyl groups, respectively [10]. Clearly, oxygen vacancies exist in bismuth-rich Bi_4_O_5_Br_2_ and Bi_5_O_7_Br, which is consistent with reported results in the literature [13,23]. The band energies of 67.79 eV and 68.84 eV belong to the peaks of Br 3d_5/2_ and Br 3d_3/2_, confirming the feature of Br^−^ for Bi_4_O_5_Br_2_ (Figure 6c) [24]. In BiOBr and Bi_5_O_7_Br, the band energy signals of Br 3d_5/2_ and Br 3d_3/2_ have also been confirmed to be 67.77 eV and 68.82 eV as well as 67.87 eV and 68.92 eV [25,26,27], respectively. Figure 6d shows that the Bi 4f spectra with two fitted peaks at band energies of 158.31 eV and 163.65 eV can be attributed to the Bi^3+^ in Bi_4_O_5_Br_2_ [17]. Nevertheless, the Bi 4f band energy of Bi_5_O_7_Br is slightly greater than that of Bi_4_O_5_Br_2_, indicating that the electron density around Bi^3+^ decreased in Bi_5_O_7_Br; this occurred because Bi^3+^ coordinates with O^2−^ and Br^−^ in Bi_4_O_5_Br_2,_ and Bi^3+^ mainly form a coordination bond with O^2−^ in Bi_5_O_7_Br as the oxygen content increases, resulting in a stronger bismuth–oxygen bond in Bi_5_O_7_Br.

### 3.4. Electronic Band Structure

**Figure 7:**
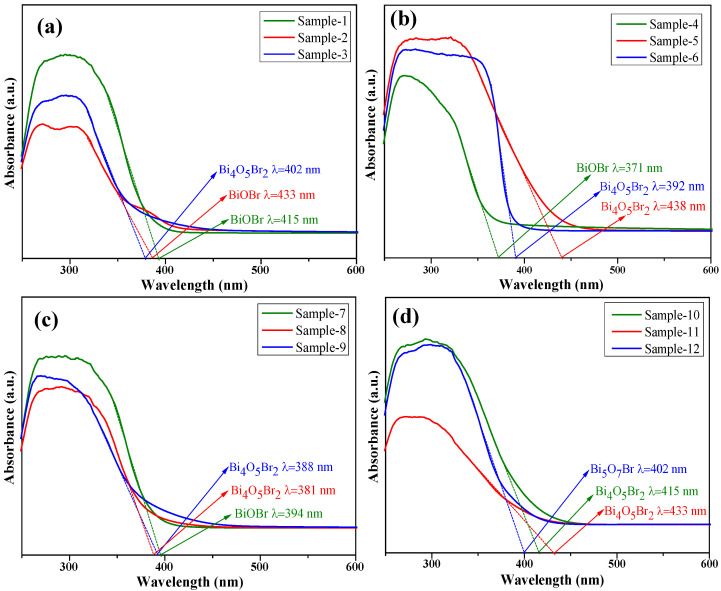
UV–Vis DRS of the samples prepared at different pH values: (**a**) pH = 5, (**b**) pH = 7, (**c**) pH = 9 and (**d**) pH = 11.

The electronic band structures of the different samples were investigated via UV–Vis DRS. Figure 7 shows the difference in optical absorption performance for the synthesised samples at various pH values and Bi:Br ratios. Notably, the light absorption edges of BiOBr, Bi_4_O_5_Br_2_ and Bi_5_O_7_Br are at approximately 433 nm (sample-2), 438 nm (sample-5) and 402 nm (sample-12) (Figure 7a–d), which reach the visible-light range (λ > 400 nm), respectively. However, the optical absorption of these samples occurs predominantly in the UV region, with only a marginal extension into the visible. Compared with BiOBr and Bi_5_O_7_Br, Bi_4_O_5_Br_2_ (sample-5) clearly exhibited the best visible-light response. Notably, the light absorption edge for Bi_4_O_5_Br_2_ (sample-11), with a value of 433 nm, is shorter than that of sample-5. This finding can be attributed to the differences in the morphology and size of Bi_4_O_5_Br_2_ caused by variations in the pH value (Figure 2, Figure 3, Figure 4 and Figure 5). The thinner and more loosely arranged the Bi_4_O_5_Br_2_ nanosheet units of the sample are, the stronger is their ability to absorb and reflect light. As shown in Figure 3c,d and Figure 5c,d, the Bi_4_O_5_Br_2_ nanosheets in sample-5 are more dispersed than those in sample-11. Therefore, the utilisation of light for sample-5 has improved.

**Figure 8:**
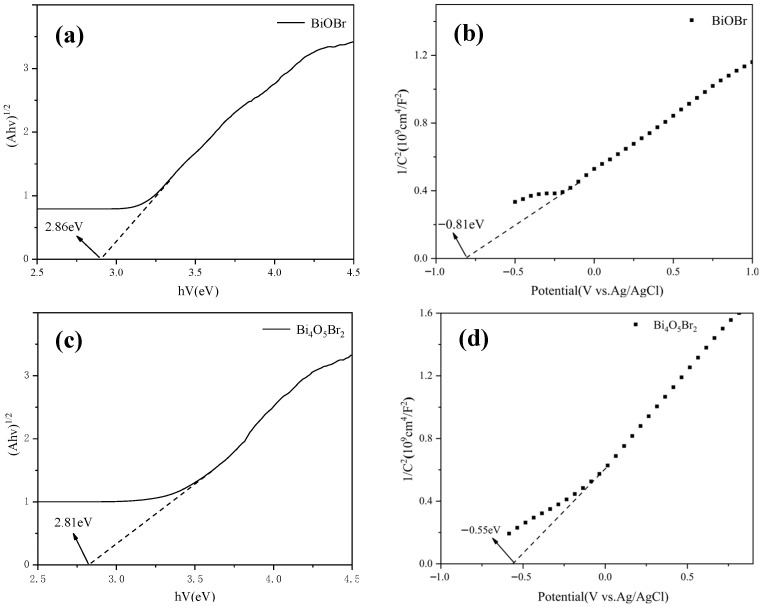

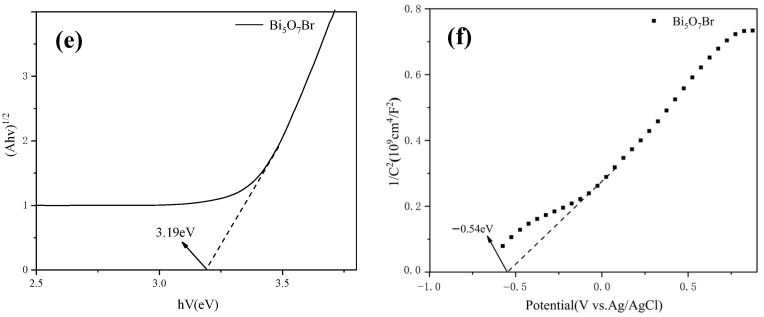
Band gap energies for (**a**) BiOBr, (**c**) Bi_4_O_5_Br_2_ and (**e**) Bi_5_O_7_Br and Mott–Schottky curves for (**b**) BiOBr, (**d**) Bi_4_O_5_Br_2_ and (**f**) Bi_5_O_7_Br.

Additionally, the band gap ($E_{g}$) of BiOBr, Bi_4_O_5_Br_2_ and Bi_5_O_7_Br can be calculated via the Kubelka–Munk transformation, according to Equation (1) [5,14]:

(1) $$\alpha hv=A{\left(hv-E_{g}\right)}^{\frac{n}{2}}$$

where α, h, v, A, and $E_{g}$ are the absorption coefficient, Planck constant, optical frequency, optical constant and band gap energy, respectively. n is a constant related to the properties of the semiconductor. Since BiOBr, Bi_4_O_5_Br_2_ and Bi_5_O_7_Br are indirect bandgap semiconductors [2,9,24], the n value was determined to be 4. Hence, the $E_{g}$ values of BiOBr, Bi_4_O_5_Br_2_ and Bi_5_O_7_Br were computed to be 2.86 eV, 2.81 eV and 3.19 eV (Figure 8a,c,e), which are similar to those reported in the literature [24,25,26,27]. Visibly, the $E_{g}$ of Bi_4_O_5_Br_2_ is smaller than that of BiOBr and Bi_5_O_7_Br. A narrower band gap benefits the efficient separation of photogenerated carriers. To confirm the energy band level, the Mott–Schottky curves of BiOBr, Bi_4_O_5_Br_2_ and Bi_5_O_7_Br are plotted in Figure 8b,d,f, where the values of the calculated flat-band potential ($E_{fb}$) for BiOBr, Bi_4_O_5_Br_2_ and Bi_5_O_7_Br are −0.81 eV, −0.55 eV and −0.54 eV with respect to Ag/AgCl, respectively. Because the potential of the conduction band ($E_{CB}$) at the bottom of the n-type semiconductor is close to $E_{fb}$, $E_{CB}$ can be calculated via Equations (2) and (3) [19,21,28]:

(2) $$E_{fb}^{\mathrm{NHE}}=E_{fb}+0.197$$

(3) $$E_{CB}=E_{fb}^{NHE}\;\mathrm{VS}.\;\mathrm{NHE}$$

where $E_{fb}^{NHE}$ is the potential of the Ag/AgCl electrode relative to the Normal Hydrogen Electrode (NHE). Hence, the $E_{CB}$ values of BiOBr, Bi_4_O_5_Br_2_ and Bi_5_O_7_Br are estimated to be −0.61 eV, −0.35 eV and −0.34 eV relative to the NHE, respectively. Additionally, the valence band potential ($E_{VB}$) can also be evaluated via Equation (4) [22,29]:

(4) $$E_{g}=E_{VB}-E_{CB}$$

**Figure 9:**
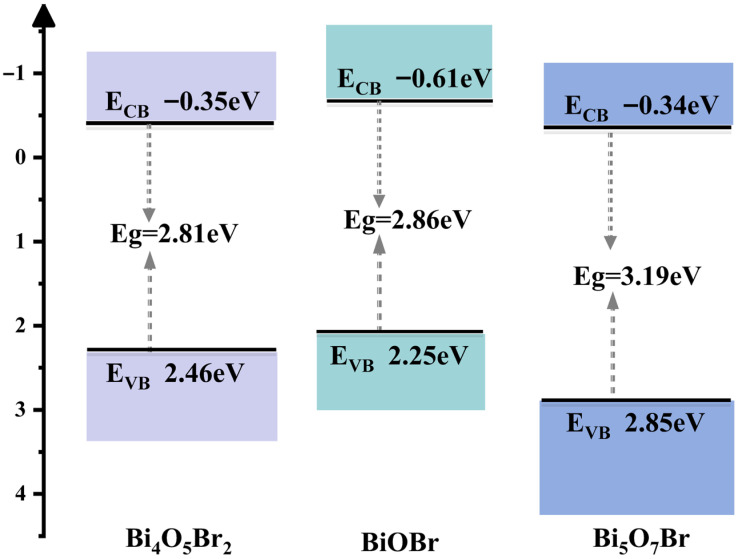
Diagram of the energy band structures of BiOBr, Bi_4_O_5_Br_2_ and Bi_5_O_7_Br.

The $E_{VB}$ values of BiOBr, Bi_4_O_5_Br_2_ and Bi_5_O_7_Br are 2.25 eV, 2.46 eV and 2.85 eV with respect to the NHE, respectively. Therefore, based on the data acquired from the previous calculations, the energy band structure graphs of BiOBr, Bi_4_O_5_Br_2_ and Bi_5_O_7_Br are plotted in Figure 9, where the more negative the $E_{CB}$ level is, the greater the thermodynamic driving force for the CO_2_ reduction reaction. Here, the $E_{CB}$ of BiOBr is smaller than that of Bi_4_O_5_Br_2_ and Bi_5_O_7_Br, but its $E_{g}$ is also greater than that of Bi_4_O_5_Br_2_. Nevertheless, the $E_{CB}$ and $E_{g}$ of Bi_5_O_7_Br are the greatest, which may have a negative impact on the photocatalytic activity for CO_2_ reduction.

### 3.5. Specific Surface Area and CO_2_ Adsorption

**Figure 10:**
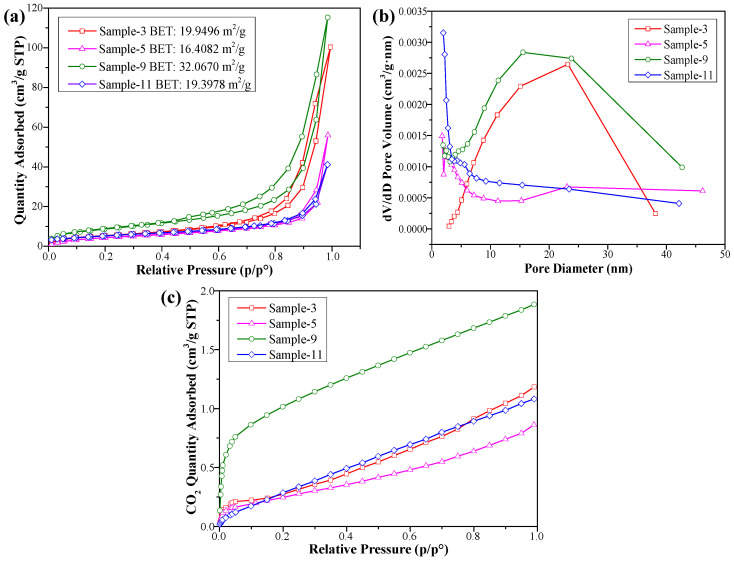
(**a**) N_2_ adsorption–desorption isotherms, (**b**) pore volume distribution curves and (**c**) CO_2_ adsorption capacity of Bi_4_O_5_Br_2_ in different samples.

To study the influence of microstructure on the active sites, the N_2_ adsorption–desorption curves of Bi_4_O_5_Br_2_ samples with various microstructures prepared at different pH values and Bi/Br ratios are shown in Figure 10. The physisorption–desorption isotherms for all the samples have an IV-shaped lag ring (Figure 10a), which reveal that the distribution of pores in Bi_4_O_5_Br_2_ is dominated by micro-mesopores [26,30]. As shown in Figure 10b, micropores dominate sample-5 and sample-11, and mesopores play a major role in sample-3 and sample-9. This structure is conducive to the reflection of light and adsorption of carbon dioxide [31,32]. Table 3 lists the BET surface properties of the different samples. Notably, sample-9 has the largest specific surface area (32.07 ± 0.28 m^2^·g^−1^) and largest pore volume (0.18 cm^3^·g^−1^) compared with the other samples. Figure 10c shows the CO_2_ adsorption curves, where the maximum adsorption capacity of CO_2_ for sample-9 is 1.88 cm^3^·g^−1^. For sample-11, the maximum adsorption value of CO_2_ is 1.08 cm^3^·g^−1^. Sample-11 clearly has the average CO_2_ adsorption capacity among the four samples (Table 3). The CO_2_ adsorption capacity is dependent on the specific surface area and number of active sites [33,34]. Here, the specific surface area (19.40 ± 0.18 m^2^·g^−1^) of sample-11 is smaller than those of samples 9 and 3 (Figure 10a), which suggests that sample-11 has relatively fewer active sites.

### 3.6. Photocatalytic CO_2_ Reduction Performance

**Figure 11:**
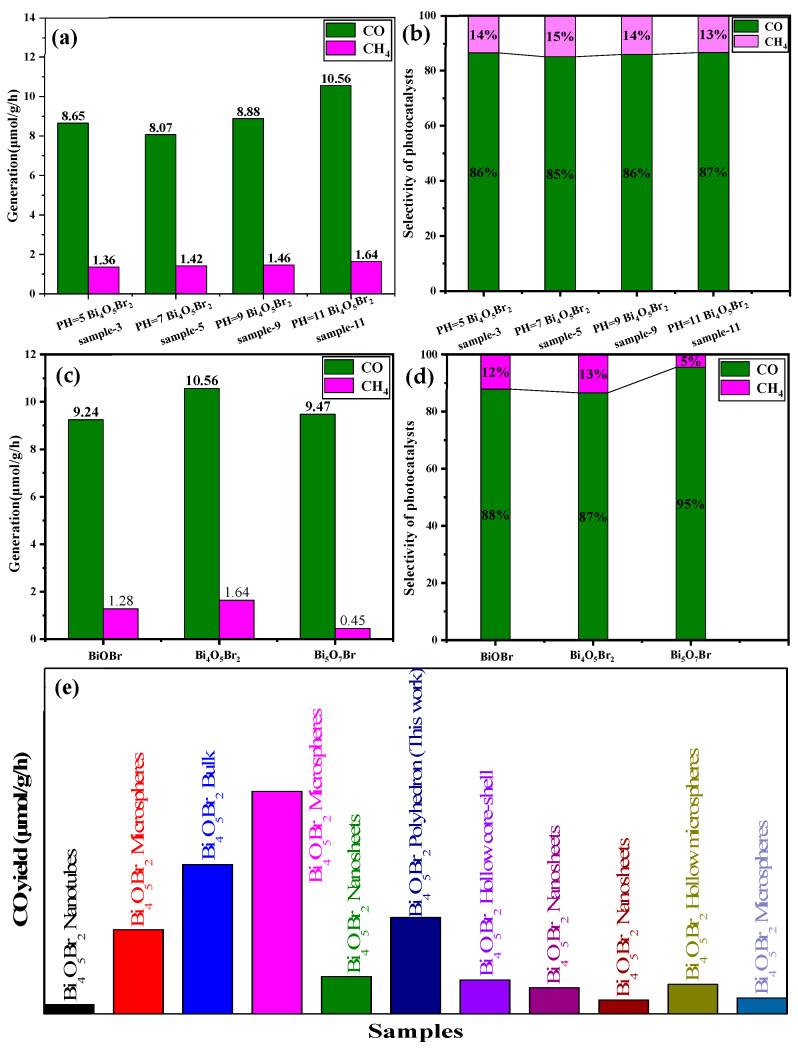
(**a**) Generation rates and (**b**) selectivity of CO and CH_4_ for Bi_4_O_5_Br_2_ synthesised at different pH values and Bi/Br ratios; (**c**) generation rates; (**d**) selectivity of CO and CH_4_ for BiOBr, Bi_4_O_5_Br_2_ and Bi_5_O_7_Br; and (**e**) comparison of CO production rates for different samples [2,8,14,22,24,41,42,43,44,45].

The photoreduction of CO_2_ by Bi_4_O_5_Br_2_ results in the production of the main product CO and the byproduct CH_4_ in this study (Figure 11a). The CO production rates of sample-3, sample-5, sample-9 and sample-11 are 8.65, 8.07, 8.88 and 10.56 μmol·g^−1^·h^−1^, respectively. The CH_4_ conversion rates of the corresponding samples were also 1.36, 1.42, 1.46 and 1.64 μmol·g^−1^·h^−1^, respectively. The CO productivity of sample-11 clearly has a maximum value of 10.56 μmol·g^−1^·h^−1^, and the photocatalytic selectivity of sample-11 for CO products with a value of 87% is greater than that of the other samples (Figure 11b). Based on the above data, sample-11 exhibited the optimal photocatalytic performance and selectivity among the four samples. Although the phase composition of the four samples is Bi_4_O_5_Br_2_ (Figure 1), the differences in photoreduction performance and selectivity of CO_2_ can be attributed to the various microstructures of the four samples (Figure 2, Figure 3, Figure 4 and Figure 5). Sample-9 presents a loose and irregular polyhedron morphology (Figure 4e,f) and has the largest specific surface area and pore volume (Table 3), which is conducive to CO_2_ adsorption, but it is not beneficial for multiple light scattering [35,36]. Therefore, sample-9 has the strongest CO_2_ adsorption capacity (1.88 cm^3^·g^−1^) and a relatively weak visible-light absorption edge (388 nm) (Figure 7c). Although sample-11 also has an irregular polyhedron morphology, the specific surface area and pore volume are smaller than those of sample-9, which leads to a lower CO_2_ adsorption capacity (1.08 cm^3^·g^−1^) and a larger visible-light absorption edge (433 nm) (Figure 7d). Consequently, the synergistic effect of the CO_2_ adsorption capacity and light absorption range results in sample-11 having the best photocatalytic performance.

Figure 11c compares the CO productivity of synthesised BiOBr (sample-2), Bi_4_O_5_Br_2_ (sample-11) and Bi_5_O_7_Br (sample-12) for the photoreduction of CO_2_, where BiOBr and Bi_5_O_7_Br have similar CO productivity values of approximately 9.24 μmol·g^−1^·h^−1^ and 9.47 μmol·g^−1^·h^−1^, respectively. However, the CO yield of both is lower than that of Bi_4_O_5_Br_2_ (10.56 μmol·g^−1^·h^−1^). This difference can be attributed not only to the differences in their electronic energy bands (Figure 9) but also to their shapes. The morphology affects the light absorption capacity and the number of active sites for CO_2_ adsorption [37]. Compared with the particle-like and bulk Bi_5_O_7_Br nanosheets [10,27], the Bi_5_O_7_Br nanosheets obtained in this study have advantages in terms of photocatalytic performance. Moreover, the energy band structure can affect the number of electronic transitions and the separation efficiency of photogenerated carriers [38]. The $E_{g}$ of Bi_4_O_5_Br_2_ is smaller than those of BiOBr and Bi_5_O_7_Br (Figure 9). However, the $E_{CB}$ of Bi_4_O_5_Br_2_ is moderate. Additionally, oxygen vacancies (OVs) can form in bismuth-abundant Bi_4_O_5_Br_2_ or Bi_5_O_7_Br (Figure 6b). Several studies have reported that bismuth-rich Bi_4_O_5_Br_2_ and Bi_5_O_7_Br are prone to form oxygen vacancies [2,22,27,39], which can further promote the separation of photogenerated carriers and the reduction of CO_2_ [27,40]. Hence, Bi_4_O_5_Br_2_ exhibited the optimal photocatalytic CO_2_ reduction performance. With respect to the selectivity of the primary product CO, that of Bi_5_O_7_Br, with a value of 95%, is greater than that of BiOBr and Bi_4_O_5_Br_2_ (Figure 11d). Nevertheless, the Bi_4_O_5_Br_2_ with the selectivity of the product CO is the lowest among them. Additionally, Figure 11e shows a comparison of the production rates of the main product CO reported in the literature for Bi_4_O_5_Br_2_. The obtained polyhedron Bi_4_O_5_Br_2_ has a certain advantage in the production rate of CO [2,8,24,41,42,43,44,45]. However, with respect to the production rate of CO, that of Bi_4_O_5_Br_2_ is also lower than those reported in several studies [14,22]. This difference is caused by the morphology and microstructure of the Bi_4_O_5_Br_2_ catalyst.

## 4. Conclusions

In this study, BiOBr, Bi_4_O_5_Br_2_ and Bi_5_O_7_Br photocatalysts were successfully synthesised via a hydrothermal method by adjusting the molar ratio of Bi:Br and the pH of the precursor solution. Owing to the effects of pH and the Bi/Br ratio, a phase transformation occurred from BiOBr→Bi_4_O_5_Br_2_→Bi_5_O_7_Br. Under acidic conditions, BiOBr can be formed easily. Under alkaline conditions, bismuth-rich Bi_4_O_5_Br_2_ or even Bi_5_O_7_Br was generated.

BiOBr presented a morphology of hollow microspheres, irregular microspheres, irregular and loose microspheres and polyhedrons. The Bi_4_O_5_Br_2_ had relatively regular and loose microspheres, irregular polyhedrons and loose and irregular polyhedrons. The Bi_5_O_7_Br exhibited a dense lamella shape. Under acidic conditions, relatively regular microspheres were dominant. Under alkaline conditions, irregular polyhedron or even nanosheet shapes were obtained.

The UV–visDRS results revealed that the light absorption edges of BiOBr, Bi_4_O_5_Br_2_ and Bi_5_O_7_Br were at approximately 433 nm, 438 nm and 402 nm, respectively, reaching the visible-light range. The band structure indicated that the $E_{g}$ of Bi_4_O_5_Br_2_ was smaller than those of BiOBr and Bi_5_O_7_Br. However, its $E_{CB}$ was moderate. Nevertheless, the $E_{CB}$ and $E_{g}$ of Bi_5_O_7_Br were the highest among the three samples.

With respect to Bi_4_O_5_Br_2_, the CO_2_ photoreduction of samples obtained under different conditions has various yields for the main product, CO, and the by-product, CH_4_. Among them, the CO productivity of sample-11 has a maximum value of 10.56 μmol·g^−1^·h^−1^_,_ and the selectivity for CO reaches 87%. This difference can be attributed to the influence of morphology and microstructure. Additionally, BiOBr and Bi_5_O_7_Br have similar CO productivity values of approximately 9.24 μmol·g^−1^·h^−1^ and 9.47 μmol·g^−1^·h^−1^, respectively, which are lower than that of Bi_4_O_5_Br_2_. Hence, Bi_4_O_5_Br_2_ exhibited the optimal photocatalytic CO_2_ reduction performance.

## Figures and Tables

**Table 1:** The number of obtained samples and the corresponding preparation conditions.

Sample Number	1	2	3	4	5	6	7	8	9	10	11	12
Bi/Br ratio	1:1	2.55:1	3:1	1:1	2.55:1	3:1	1:1	2.55:1	3:1	2:1	4:1	8:1
PH value	5	5	5	7	7	7	9	9	9	11	11	11

**Table 2:** Chemical compositions and atomic ratios of three samples.

Sample Number	Element Type and Atomic Percent			
	Bi (at.%)	O (at.%)	Br (at.%)	Bi/Br (Ratio)
Sample-1	14.55	74.30	11.15	1.3:1
Sample-11	23.97	70.57	5.46	4.4:1
Sample-12	26.27	70.35	3.38	7.8:1

**Table 3:** Surface properties of the different samples.

Samples	BET Surface (m^2^·g^−1^)	Pore Volume (cm^3^·g^−1^)	Pore Diameter (nm)	CO_2_ Adsorption Capacity (cm^3^·g^−1^)
Sample-3	19.95 ± 0.12	0.16	26.6 ± 5.6	1.18
Sample-5	16.41 ± 0.35	0.09	20.9 ± 1.1	0.86
Sample-9	32.07 ± 0.28	0.18	20.5 ± 2.7	1.88
Sample-11	19.40 ± 0.18	0.06	13.78 ± 0.89	1.08

## Data Availability

The original contributions presented in this study are included in the article. Further inquiries can be directed to the corresponding author.
